# Community Pharmacists' Perceptions about Pharmaceutical Care of Traditional Medicine Products: A Questionnaire-Based Cross-Sectional Study in Guangzhou, China

**DOI:** 10.1155/2016/7801637

**Published:** 2016-03-15

**Authors:** Xi Chen, Carolina Oi Lam Ung, Hao Hu, Xiaodan Liu, Jing Zhao, Yuanjia Hu, Peng Li, Qing Yang

**Affiliations:** ^1^State Key Laboratory of Quality Research in Chinese Medicine, Institute of Chinese Medical Sciences, University of Macau, Macau; ^2^State Key Laboratory of Hydraulics and Mountain River Engineering, Sichuan University, Chengdu, Sichuan 610000, China

## Abstract

This study aimed to investigate community pharmacists' perceived responsibility, practice behaviors, knowledge, perceived barriers, and improvement measures towards provision of pharmaceutical care in relation to traditional medicine (TM) products in Guangzhou, China. A self-completion questionnaire was used to survey licensed pharmacists working at community pharmacies. This study found that the community pharmacists in Guangzhou, China, were involved in the provision of TM products during their daily practice but only provided pharmaceutical care in this area with a passive attitude. Extrinsic barriers such as lack of scientific evidence for the safety and efficacy of TM products and unclear definition of their roles and responsibilities were highlighted while intrinsic factors such as insufficient TM knowledge were identified.

## 1. Introduction

Traditional medicine (TM), also known as complementary or alternative medicine, is widely adopted around the world to different extent depending on the form of the health care system. The market for TM products is substantial. The output of Chinese materia medica was estimated at US$83.1 billion in 2012, an increase of more than 20% from the previous year [1]. The European Information Center of Complementary and Alternative Medicine indicated that more than 100 million of the European population consumed TM products and higher figures could be seen in Africa, Asia, and Australia [2]. Out-of-pocket expenditure on natural products was US$14.8 billion in the US in 2008 [3]. In Canada, natural health products are worth over $400 million annually on average [4]. One of the reasons for the increasing demand is the purpose of using TM products which has shifted beyond health promotion and health maintenance in recent years. Many people are now using TM products to manage their ailments without consulting any health care professionals, a practice known as self-medication. Moreover, the costs of health care and the prevalence of chronic diseases continue to rise especially among aging population. Being comparatively affordable and generally accessible, self-medication with TM products has now become a very popular form of health care practice.

This is especially the case in China where traditional Chinese medicine (TCM) is a typical form of TM and an integral part of the health care system. TCM products are commonly used alongside conventional medicines at every level of the health care in China to various extents [1]. A large household survey in both urban and rural China showed that 54% of the population preferred Western medical treatment, 25% preferred TCM/Western integrated treatment, 12% preferred TCM treatment only, 5% preferred Western medicine for acute conditions and TCM for chronic conditions, and 1% preferred Western medicine for diagnosis and TCM for treatment [5]. Due to historical and cultural reasons, TCM products mostly in the form of proprietary Chinese medicines (PCMs) have been widely recognized as highly effective during self-medication and the demand for them is always on the rise [6].

The combination of self-medication and TM products has magnified the reasons for safety concerns. Self-medication for one ailment may be straightforward. However, considering concurrent disease states, overavailability of medicine choices, and mixed information about the products available over the counter, professional supervision is warranted to ensure correct recognition of health problems, right choice of medicine, appropriate dosing and duration of treatment, and raised awareness about possible adverse drug effects (ADEs)/adverse drug reactions (ADRs), interactions, and precautions [7, 8]. For TM products, like conventional medicines, there are also potential risks of ADRs even when used according to guidance. However, people do not usually have the same level of respect for TM products. It is still a common perception, or rather a misconception, that TM products are made of herbal ingredients and are therefore safe to use. In China, out of the 1.33 million case reports of ADE/ADR received by the National ADR Monitoring Center received in 2014, TCM drugs represented around 17.3% [9].

Depending on the pharmacokinetic properties (absorption, distribution, metabolism, and elimination) and pharmacodynamics behaviors (synergistic or antagonistic), the extent and the way TM products interact with conventional medicines and other disease states are numerous and can possibly lead to disastrous consequences. For instance, the herb* dang gui (Angelica sinensis)*, which is commonly consumed on a regular basis for health promotion especially by menopausal women, can potentiate the anticoagulant effects of blood thinning agents. Concomitant use is contraindicated as this can result in widespread bruising [10, 11]. Moreover, formulations of TM products are usually complicated, and the ingredients and mechanisms of action thereof are not usually fully disclosed or acknowledged [12]. For patients to use over-the-counter TM products without the supervision of health care professionals, necessary patient monitoring and drug safety assurance can easily be compromised.

In light of the challenges of safe and appropriate use of TM products, it has been suggested that pharmacists should play a role in helping patients make safe and informed choices about TM products for self-medications through appropriate pharmaceutical care. Pharmaceutical care as suggested by Helper and Strand in 1990 is the “provision of drug therapy for the purpose of achieving definite outcomes that improve a patient's quality of life” [13]. In many countries, TM products for self-medications are mostly OTC medicines available in the pharmacies which are supervised by pharmacists. For patients who want TM products at community pharmacy, pharmacists are probably the only health care professionals who are in position to intervene. It is also a general belief that the education and training in pharmacy school ensure the pharmacist acquires necessary knowledge to make appropriate comment on the choice of TM products [14]. After all, the role of pharmacists is the gate keeper for drug safety as recommended by the World Health Organization [15]. Consumers may also have the perception that pharmacists are competent enough to prevent, identify, and resolve drug-related problems (DRPs) about TM products the way they practice with conventional medicines [16]. There is also evidence showing that patients have firm trust in pharmacists and their knowledge about TM products [17, 18].

In reality, the dilemma is that pharmacists are trained to practice primarily with conventional medicine. The training of TM is not necessarily included in the syllabus of the pharmacy schools or required for professional registration. The general lack of evidence-based information about TM also makes it difficult for pharmacists to determine the appropriateness, the safety and the efficacy of TM products even with the knowledge of pathophysiology and pharmacology, and skills to interpret and evaluate results of clinical studies. Professionally, ethically, and legally, the role of pharmacists in relation to TM products is to be determined and many studies are being carried out worldwide. For example, a pilot study with Australian community pharmacists found that they had varied views about complementary and alternative medicine and there was a general shortage of knowledge to support related pharmaceutical care [19]. According to a key informant study in Canada, stakeholder leaders suggested that community pharmacist should take responsibility for safety monitoring for natural health products but did not have sufficient knowledge [20].

In China, the prevalence of self-medication is reportedly high which highlighted the important value of community pharmacists in ensuring patients' drug safety. Although community pharmacists were primarily charged with duties of compounding and dispensing medications according to doctors' prescriptions, there was great determination to improve the quality of pharmaceutical care. In 2007, a revised version of “Guidelines for Good Pharmacy Practice (GPP)” was adopted which detailed the role of pharmacists in community settings and mandated the provision of pharmaceutical care [21]. Pharmaceutical care at community level in China was developing and many challenges could be expected especially in the area of self-medication with TM products [22].

This study aimed to investigate this issue based on community pharmacists' perceived responsibility, practice behaviors, knowledge, perceived barriers, and improvement measures towards the provision of pharmaceutical care in relation to TM products in Guangzhou, China. It is expected that results of this study will contextualize the current findings and facilitate decision-making in improving patient safety regarding TM products for other areas in China and other countries.

## 2. Methods

This study used a paper-form questionnaire to collect first-hand data about community pharmacists' opinions on TM products available OTC in pharmacies. The survey design was reviewed and approved by the Ethics Committee of the Research Committee at the University of Macau.

### 2.1. Study Target

In this study, Guangzhou was chosen as the target city for several reasons. Firstly, Guangzhou has a prolonged history of TM usage. Early in the 19th century, there were already four-time honored Chinese medicine enterprises in Guangzhou and imported TM products were already available in the market [23]. Secondly, Guangzhou has a high utility rate of TM products (including PCMs) with consumption ranked the highest across the country. Thirdly, the number of registered pharmacists in Guangzhou is the largest in Guangdong province [24]. It is anticipated that the findings of this study are representable to provide informative results about pharmaceutical care in relation to TM products in China.

This study only focused on community pharmacists who worked at community pharmacies for two major reasons. Firstly, compared with the pharmacists at community health centres and stations, pharmacists at community pharmacies were subject to greater autonomy to recommend TM products and to provide pharmaceutical information. Secondly, the number of pharmacists at community health centres and stations is very limited. This study was carried out in all 11 administrative districts in Guangzhou. According to the information provided on the official website of the Guangzhou Municipal Food and Drug Administration, it was found out that there were 2,116 registered community pharmacies in Guangzhou. The pharmacies were cross-checked through Baidu Internet map (http://map.baidu.com/) to find out the exact location, which gave a result of around 2,200 community pharmacies eventually. To determine the sample size, the margin of error (standard error) was set to be 0.05 with 95% confidence interval. With an added contingency of 10% for nonresponse and inappropriate responses, the final sample size was set to be 270 pharmacies representing around 12% of the total number. In order to obtain stratified samples representing different social and economic environments, questionnaires were sent to 12% of the pharmacies in each district. The community pharmacies in each district were numbered at first and then randomly selected. The whole survey was carried out from October to November 2014.

### 2.2. Sampling and Data Collection

One of the authors was the investigator who personally visited the pharmacies. The licensed pharmacist in charge of provision of pharmaceutical care at each community pharmacy studied was targeted as the survey respondent who was identified by inquiry or badge observation. Before the formal survey started, verbal informed consent was obtained from the respondents. A standardized working process was then carried out by the investigator: (1) self-introduction; (2) systemic explanation of research background and objectives as indicated on the cover page of questionnaire; (3) declaration of academic and anonymous nature of this survey; and (4) reconfirmation of respondents' participation. Whenever respondents made queries about the study, the investigator would provide more specific information. The questionnaires were completed by respondents without any intervention. All of the questionnaires were distributed directly to the respondents and collected on site during the same visit.

### 2.3. Measurements

A structured questionnaire was developed based on thorough review of past literature [19, 20, 25–41]. Then, a pilot survey of the questionnaire was carried out with 30 community pharmacists. Finally, necessary modifications were made based on the comments received from the pilot survey. This prime focus of this study was limited to PCMs to facilitate respondents' understanding of the questionnaire.

The questionnaire consisted of five sections. The first section collected demographic data of the respondents such as gender, age, the number of years practicing as pharmacist, highest qualification related to pharmacy, type of pharmacy respondents practiced, and personal experience of using TM products.

In the second section, respondents were questioned about their perceived responsibilities with regard to TM products ethically and legally, and whether they should sell or recommend TM products to customers. Five choices of answers were provided to answer each single-answer question (“Strongly Disagree,” “Disagree,” “Neutral,” “Agree,” and “Strongly Agree”). The third section consisted of 10 questions and was designed to assess respondents' basic knowledge about TM products commonly available in community pharmacists. Based on the popularity of the products and the most commonly discussed issues about PCMs in the literature, the questions were formulated to cover drug safety, ADRs, and drug interactions, as well as precautions specific to concomitant use with conventional medicines, target groups, and concurrent disease.

There were two parts in the fourth section. Respondents were asked about the way they provided pharmaceutical care in relation to TM products. In the first part, respondents were questioned about the frequency of selling TM products, counseling consumers about correct usage of the products, ADRs, and drug interactions. Four choices of answer were provided to answer each single-answer question (“Never,” “Only upon customers' enquiries,” “Whenever deemed appropriate,” and “To all patients”). The second part is a multianswer question trying to find out the source of information respondents used when counseling patients about TM products. In the last section, respondents were asked about their perceived barriers and improvement measures towards the provision of pharmaceutical care in relation to TM products.

### 2.4. Data Analysis

SPSS 19.0 for Windows was used for inputting and analyzing data. Descriptive analysis and Chi-square test were applied in data analysis. The demographics, attitudes, perceived responsibility, knowledge, and practice behaviors of the respondents were analyzed by descriptive analysis. In addition, Chi-square test was used to analyze the significant effect of five factors on the practice behaviors, perceived responsibility, perceived barriers, and improvement measures. The five factors included gender, age, years of practice as pharmacist, highest qualification in relation to pharmacy, and the type of pharmacy they worked at. The factors indicated statistically significant differences at the *P* < 0.05 level.

## 3. Results

### 3.1. Demographics of the Respondents

Out of 270 questionnaires distributed, 18 were rejected and 252 were received giving a return rate of 93.3%. Due to incompletion, failure to meet completion requirements, or non-single-choice answer to the knowledge questions, 21 of the returned questionnaires were excluded, resulting in 231 questionnaires for further analysis. The demographics of the respondents were summarized in Table 1. Results showed that female pharmacists numbered at 151 and accounted for 65.4% of the respondents in two major age groups: 31~40 years (29.9%) and 41~50 (35.9%); the years of practice were mainly in the ranges of 5~10 years (30.7%) and 11~20 years (41.6%). In terms of the highest qualification in relation to pharmacy, the majority (68.8%) of the pharmacists obtained a junior college degree or below. As for type of pharmacy, nearly 60% (59.3%) of the respondents worked in chain pharmacies.

### 3.2. Respondents' Perceived Responsibility in relation to TM Products

Table 2 shows that 39% of the respondents agreed or strongly agreed that they should recommend and sell TM products while 50% were neutral. Regarding ethical responsibility of safeguarding drug safety of TM products, 41% of the respondents agreed or strongly agreed while 37% remained neutral and 21% disagreed or strongly disagreed. In terms of legal responsibility, 31% of the respondents agreed or strongly agreed that they were legally responsible for the drug safety of TM products while 46% remained neutral and 23% disagreed or strongly disagreed.

According to Chi-square test (see Table 3), there are statistically significant differences in the results of the 3 questions: (1) age on the opinions about whether pharmacists should recommend or sell TM products (*X* ^2^ = 26.294  *P* = 0.030); (2) age on the opinions about ethical responsibility (*X* ^2^ = 32.834  *P* = 0.009); (3) highest qualification in relation to pharmacy on the opinions about ethical responsibility (*X* ^2^ = 18.181  *P* = 0.017).

### 3.3. Respondents' Knowledge of TM Products

For the 10 knowledge questions, the average score by the respondents was 5.9. Less than 2% answered all the 10 questions correctly. 40% of the respondents scored 5 or less than 5 points.  Table 4 listed the results of each question. Question (1) was about general safety of TM products and less than 65% of respondents knew that TM products were not always safe and were associated with side effects. For Question (2) regarding concomitant use with conventional medicines, 42% acknowledged that not all OTC Chinese medicine and Western medicine can be taken at the same time. Questions (3), (6), (7), and (10) asked about drug interactions of TM products and there were mixed results. While about 80% got two of these questions right (Questions (3) and (6)), only around 60% of respondents answered the other two questions correctly (Questions (7) and (10)). Questions (4), (5), and (9) were concerned about precautions specific to disease state and target population. Around 33% of respondents were correct about the precautions taken by patients with G6PD deficiency in relation to TM products. 92.6% knew that pregnant women should not take “Niu Huang Jie Du Pian.” This TM product can cause miscarriage and is therefore contraindicated in pregnant women. More than 50% of respondents were not aware of the risk of heavy metals in “Bo Ying Compound” for infants and toddlers. Question (8) was concerned about ADRs and only 36% of respondents were aware of the ADRs of a commonly used herb* Glycyrrhiza glabra*.

It was found that 94.4% of respondents had learnt about TM products of which 58% learned about TM in pharmacy school and 52.0% and 49.0% acquired knowledge about TM through continuing professional education and self-learning, respectively. Some of them also learned about TM through employer-sponsored training (27.1%) or manufacturer-provided training (27.1%).

For respondents' information sources about TM products, 57.0% and 67.0% indicated peer-reviewed medical journals and medical/drug-related reference books as the major sources of information about TM products, respectively. In addition, 43.7% of respondents also used databases such as Wanfang Data, PubMed, and Medline. Professional seminars, conferences, or lectures (38.1%), word of mouth (e.g., friends and family) (33.3%), and World Wide Web (e.g., Google) (30.3%) were the next popular sources of TM product information.

### 3.4. Respondents' Practice Behaviors in relation to Pharmaceutical Care of TM Products

Overall, the respondents provided pharmaceutical care in relation to TM products only to some level (see Table 5). There were five types of practice behaviors assessed in the questionnaire including “selling TM products,” “discussing the use of TM products,” “advising on appropriate dosing and duration of treatment,” “advising on possible ADRs,” and “advising on important interactions.” As shown in Table 5, 19% of respondents would try to sell TM products to all customers while 79% would sell TM products whenever deemed appropriate and upon consumers' requests. Around 50% of respondents would proceed to further discussion with the consumers on the use, dosing and duration of treatment, and ADRs of the TM products “whenever deemed appropriate.” Between 26 and 38% would do that “only upon customers' enquiries.” Regarding advising consumers on interactions of TM products, 44% and 42% chose “whenever deemed appropriate” and “only upon customers' enquiries,” respectively.

Results of statistical analysis in Table 6 showed that there were numerous statistically significant differences in several aspects of respondents' practice behaviors in relation to pharmaceutical care of TM products: (1) gender on the frequency of selling TM products in the pharmacy (*X* ^2^ = 9.909, *P* = 0.013); (2) age on the frequency of advising customers about common ADRs of TM products (*X* ^2^ = 26.132, *P* = 0.004); (3) highest pharmacy-related qualification on the frequency of selling TM products in pharmacy and frequency of advising customers about the use and common ADRs of TM products (*X* ^2^ = 18.868, *P* = 0.004; *X* ^2^ = 17.405, *P* = 0.009; and *X* ^2^ = 18.708, *P* = 0.000, resp.).

### 3.5. Respondents' Perceived Barriers and Improvement Measures towards Pharmaceutical Care in relation to TM Products

A number of perceived barriers were identified by the respondents which included in the order of importance “lack of scientific evidence about TM products” (66.2%), “ambiguity of the professional role of pharmacist” (52.8%), and “lack of appropriate professional knowledge” (50.2%). “Lack of time” (39.4%) and “unwillingness of customers to seek pharmaceutical care from pharmacists” (30.3%) were the other two challenges affecting the provision of pharmaceutical care in relation to TM products.

Out of the 6 measures for improving pharmaceutical care in relation to TM products as listed in Table 7, training about TM products was mostly needed to enhance pharmacists' ability to counsel patients about TM products (94.4%) followed by public education to promote the professional role of pharmacists (92.6%) and legislation to clearly indicate the role of pharmacists in relation to TM products (86.6%). Respondents also found that standards of practice to highlight the role of pharmacists in relation to TM (78.4%) and availability of scientific-based information about TM products (62.8%) would further support their pharmaceutical care with TM products. However, for perceived barriers and improvement measures, there was no significant statistics difference among respondents' demographics.

## 4. Discussion

As adoption of TM and the use of the products are deeply rooted in the Chinese culture, TM products were widely provided at community pharmacies in China, where community pharmacists were expected to provide pharmaceutical care to improve consumers' proper use of TM products. However, the study results revealed that community pharmacists in China did not routinely recommend TM products to the consumers. They also held a passive attitude in their practice and discussed the use, dosing and duration of treatment, ADRs, and important interactions and precautions only when deemed necessary or upon customers' request. The results are similar to that of a study conducted in Saudi Arabia in 2013, which found out that few pharmacists initiated communication about TM products with all patients [39]. Other studies also showed that although there were discussions about drug use of TM products, two-way communication was relatively rare between the pharmacists and patients [34]. For such kind of passive perception of respondents, multiple reasons need to be discussed in the following.

Firstly, based on survey findings, it appeared that most pharmacists were concerned by the lack of scientific evidence about TM products. This observation accorded with the findings in existing literature which recognized lack of understanding of the safety use of TM products as an important inhibiting factor in pharmacists' practice [19]. Although TCM is well integrated in the Chinese health care system and plays a critical role in improving public health, the theories and methodologies are not recognized and understood by pharmacists who mostly received education of Western medicine. Without the evidence-based information justifying the safety and efficacy, it is difficult for pharmacists to determine which TM products really work and how for their patients. Moreover, as TCM and Western medicine are based on different philosophies and theories, it can also become a matter of ethical consideration for pharmacists to recommend a combination of therapy when the safety and efficacy are in doubt.

Secondly, the lack of initiative in the community pharmacists' pharmaceutical care of TM products could also be explained by the management system of pharmacists in China. Under the registration scheme, two types of pharmacists are registered and licensed to practice: pharmacists for Western medicines and pharmacists for TCM. Theoretically, each of the two types of pharmacists is independently responsible for drug safety of either conventional medicine or TM. While it is common for pharmacies to have both types of pharmacists on site, there is no bridging mechanism for information exchange to evaluate consumers who take TM products and conventional medicine concurrently. There is a lack of practice standards which require pharmacists for Western medicine to routinely respond and practice with respect to TM products. According to the Licensed Pharmacist Examination Implementation Measures, there is clear indication that pharmacists of these two types should practice within their scope of expertise and no implication of crossing over when providing pharmaceutical care [42]. The results of the respondents' perceived responsibility in this study can further substantiate this argument. Most of the pharmacists in the study were neutral or even negative about their legal and professional responsibility in the area of TM products which was a clear indication of ambiguity in their perception. The integration of TCM and conventional medicine warrants a need for an improved understanding of the benefits and risks associated with using different forms of treatment concurrently. This gap of knowledge should be addressed duly by the registration body as a top priority. At the same time, firm recommendation of required competencies suggested by professional bodies would help both the pharmacists and the public to appreciate the level of pharmaceutical care that is expected from the profession.

Thirdly, although a significant proportion of respondents reported that they had received training about TM products, the findings indicated that pharmacists' knowledge about TM products was generally inadequate, which is similar to the findings in other studies [36, 38]. It is also worth notice that studies have found that pharmacists' knowledge of herbal products also varied widely from product to product [43]. The diversity in TM products makes it even harder for pharmacists to keep up with the adequacy of knowledge. As shown in other studies, insufficient knowledge negatively affected the level of confidence in answering patient inquiries, which could help explain the passive approach the pharmacists showed with regard to TM products in this study [25, 44]. In particular, for pharmacists of Western medicine in China, education about TM could come from undergraduate training. However, the extent to how TM is taught and integrated varied widely among pharmacy schools. Unlike some European countries which included phytomedicines as a compulsory subject of pharmacy teaching, there is no standardized approach to TM teaching in pharmacy schools in China. There is a general perception of the consumers about the level of TM related knowledge that pharmacists acquire. What really happens is that TM related curriculum is highly limited in many pharmacy schools and pharmacists are trained to practice primarily in the field of conventional medicine.

Finally, it should be noted that respondents' perceptions were significantly different among their age and highest qualification related to pharmacy. Our analysis found that younger respondents were more positive about their professional responsibility to provide pharmaceutical care about TM products and had more related practice. Compared with the respondents who graduated from junior college or below, the respondents with bachelor degree or above had more positive perceptions about their professional responsibility and practice of pharmaceutical care for TM products. Literature had indicated that higher education can increase pharmacists' recognition of professional identity and willingness to practise pharmaceutical care [45]. As the Chinese education department and universities had begun to take great efforts to reform its pharmacy education system in the past decade [46], it could expect that young pharmacist who were educated through a more improved education system should show more open and active attitude to pharmaceutical care of TM products. Such kind of findings highlighted the importance of increasing TM teaching in pharmacy education to promote pharmaceutical care of TM [47]. Moreover, it implies that continuous training after university education would be effective to increase community pharmacists' acceptance and practice of pharmaceutical care of TM products.

This study has several limitations that should be addressed in the future study. Firstly, this study only focused on Guangzhou. Considering the social-economic complexity and segmentation of health system across China, further research could expand into larger areas to verify our findings and obtain additional information. Secondly, this study only investigated purely from the perspective of community pharmacists without taking into consideration other contributing factors such as the financial incentives. As community pharmacists' practice has been shown to be shaped by consumers' expectations and behavior, future exploration from the view of customer is warranted.

## 5. Conclusion

This study found that the community pharmacists in Guangzhou, China, were involved in the provision of TM products during their daily practice but did not fully realize the potential to expand their roles in relation to drug safety of TM products and only provided pharmaceutical care in this area with a passive attitude. Extrinsic barriers such as lack of scientific evidence for the safety and efficacy of TM products and unclear definition of their roles and responsibilities were highlighted while intrinsic factors such as insufficient TM knowledge were identified. Future studies should try to collect opinions from other stakeholders in order to better facilitate advancement of pharmaceutical care standards for pharmacists in the area of TM products.

## Figures and Tables

**Table 1:** Respondent demographic characteristics and practice setting (*N* = 231).

Item	*n* (%)
Gender	
Male	80 (34.6%)
Female	151 (65.4%)
Age	
21–30	42 (18.2%)
31–40	69 (29.9%)
41–50	83 (35.9%)
51–60	36 (15.6%)
≥61	1 (0.4%)
Number of years practicing as pharmacist	
<5	48 (20.8%)
5–10	71 (30.7%)
11–20	96 (41.6%)
>20	16 (6.9%)
Highest qualification related to pharmacy	
Junior college or below	159 (68.8%)
Bachelor	67 (29.0%)
Master	5 (2.2%)
Pharmacy type	
Chain pharmacy	137 (59.3%)
Independent pharmacy	94 (40.7%)

**Table 2:** Respondents' perceived responsibility in relation to TM products (*N* = 231).

Questions	*n* (%)				
	Strongly disagree	Disagree	Neutral	Agree	Strongly agree
(1) Do you think pharmacists should promote or dispense TM products?	3 (1%)	22 (10%)	74 (50%)	74 (32%)	16 (7%)
(2) Ethically, do you think pharmacists are responsible for the drug safety of TM products?	3 (1%)	46 (20%)	86 (37%)	68 (30%)	28 (12%)
(3) Legally, do you think pharmacists are responsible for the drug safety of TM products?	20 (9%)	33 (14%)	67 (29%)	67 (29%)	5 (2%)

**Table 3:** Chi-square test of respondents' perceived responsibility in relation to TM products (*N* = 231).

Role perception	Gender	Age	Number of years practicing as pharmacist	Highest qualification related to pharmacy	Type of pharmacy
	*P*	*P*	*P*	*P*	*P*
Ethically, do you think pharmacists are responsible for the drug safety of TM products?	0.255	0.095	0.091	0.017^*∗*^	0.420
Legally, do you think pharmacists are responsible for the drug safety of TM products?	0.468	0.009^*∗*^	0.550	0.238	0.429
Do you think pharmacists should promote or dispense TM products?	0.662	0.030^*∗*^	0.827	0.052	0.965

^*∗*^ *P* < 0.05.

**Table 4:** Respondents' results for each of the 10 knowledge questions regarding TM products (*N* = 231).

Questions	*n* (%)		
	Choices of answer		
	Correct	Uncertain	Incorrect
(1) Most TM products consist of natural ingredients originated from animals, plants or minerals and are safe to use with no side effects.	23 (10.0%)	58 (25.1%)	150 (64.9)^**∗**^
(2) OTC Chinese medicine and western medicine can be taken at the same time.	64 (27.7%)	69 (29.9%)	98 (42.4%)^**∗**^
(3) Pharmacists should recommend patients who have always been using TM products composing angelica, ginkgo, garlic or salvia and anti-coagulant agents to cease the use of TM products promptly as concurrent use may prolong coagulation timer and increase the risk.	182 (78.8%)^**∗**^	34 (14.7%)	15 (6.5%)
(4) Customers suffering from G6PD deficiency should avoid using the products composing menthol, pearl powder, berberine, honeysuckle or bezoar.	77 (33.3%)^**∗**^	76 (32.9%)	78 (33.8%)
(5) Pregnant women can take Chinese proprietary medicine “Niu Huang Jie Du Pian”.	11 (4.8%)	6 (2.6%)	214 (92.6%)^**∗**^
(6) Concurrent use of Chinese proprietary medicine composing bile crisp such as “Liushen pills” with digitalis medicines may increase the risk of digitalis toxicity and should therefore be avoided.	187 (80.9%)^**∗**^	30 (13.0%)	14 (6.1%)
(7) Traditional Chinese medicine and OTC Chinese medicine such as honey suckle, and so forth, can be used in combination with fungi preparations like Lactasin preparations, for it can enhance the activity of fungi preparation.	32 (13.8%)	69 (29.9%)	130 (56.3%)^**∗**^
(8) TM products composing of Glycyrrhiza glabra should be used with caution because it can cause sodium retention affecting blood pressure, stimulate the mucus lining of the stomach worsening gastric ulcer and increase the breakdown of sugar increasing the risk of hypoglycaemia.	107 (46.3%)	40 (17.3%)	84 (36.4%)^**∗**^
(9) Chinese proprietary medicines such as “Bo Ying compound” which are marketed for children's use usually compose of heaving metals such as clinnabar and lead, and should therefore be avoided in infants and toddlers to avoid heavy metal toxicity.	101 (43.7%)^**∗**^	55 (23.8%)	75 (32.5%)
(10) Concurrent use of Chinese proprietary medicine “Liu Wei Di Huang Wan” can enhance the efficacy of conventional medicines such as aluminum hydroxide or aminophylline.	21 (9.1%)	69 (29.9%)	141 (61.0%)^**∗**^

^**∗**^The correct answer.

**Table 5:** Respondents' practice behaviors in relation to pharmaceutical care of TM products (*N* = 231).

Items	*n* (%)
(1) How often do you sell TM products in the pharmacy?	
Never	4 (2%)
Whenever deemed appropriate	123 (53%)
Only upon customers' requests	60 (26%)
To all customers	44 (19%)
(2) How often do you discuss the use of TM products with your customers?	
Never	3 (1%)
Whenever deemed appropriate	120 (52%)
Only upon customers' requests	82 (36%)
To all customers	26 (11%)
(3) How often do you advise customers about appropriate dosing and duration of treatment with TM products?	
Never	3 (1%)
Whenever deemed appropriate	130 (57%)
Only upon customers' requests	77 (33%)
To all customers	21 (9%)
(4) How often do you advise customers about common side effects of TM products?	
Never	5 (2%)
Whenever deemed appropriate	118 (51%)
Only upon customers' requests	87 (38%)
To all customers	210 (9%)
(5) How often do you advise customers about important interactions between TM products and conventional medicines?	
Never	14 (6%)
Whenever deemed appropriate	102 (44%)
Only upon customers' requests	96 (42%)
To all customers	19 (8%)

**Table 6:** Statistical analysis of respondents' practice behaviors in relation to pharmaceutical care of TM products (*N* = 231).

Respondents' practice behaviors in relation to pharmaceutical care of TM products	Gender	Age	Number of years practicing as pharmacist	Highest qualification related to pharmacy	Type of pharmacy
	*P*	*P*	*P*	*P*	*P*
(1) How often do you sell TM products in the pharmacy?	0.013	0.610	0.762	0.004^*∗*^	0.351
(2) How often do you discuss the use of TM products with your customers?	0.074	0.563	0.922	0.009^*∗*^	0.229
(3) How often do you advise customers about appropriate dosing and duration of treatment with TM products?	0.147	0.190	0.597	0.108	0.459
(4) How often do you advise customers about common side effects of TM products?	0.147	0.004^*∗*^	0.126	0.000^*∗*^	0.238
(5) How often do you advise customers about important interactions between TM products and conventional medicines?	0.234	0.061	0.268	0.430	0. 212
(6) What is/are your source(s) of information when discussing about TM products with customers? (multiple choices)	0.529	0.162	0.059	0.062	0.059

^*∗*^ *P* < 0.05.

**Table 7:** Respondents' perceived improvement measures towards the provision of pharmaceutical care in relation to TM products (*N* = 231).

Item	*n* (%)
(A) Strengthen the public education to promote the professional role of pharmacists	214 (92.6%)
(B) Strengthen the training in relation to TM products for pharmacists	218 (94.4%)
(C) Establish/revise legislation to highlight the role of pharmacists in relation to TM	200 (86.6%)
(D) Establish/revise standards of practice to highlight the role of pharmacists in relation to TM	181 (78.4%)
(E) Increase the availability of scientific-based information about TM products for pharmacists	200 (84.4%)
(F) Ensure appropriate human resources to allow pharmacists provide all the necessary pharmaceutical care to customers in relation to TM products.	145 (62.8%)
(G) Others (please specify)	2 (0.9%)
